# Supplementary material for: The discovery of novel noncoding RNAs in 50 bacterial genomes
Source: Nucleic Acids Res. 2024 Apr 22;52(9):5152–65. doi: 10.1093/nar/gkae248 (PMC11109978; doi:10.1093/nar/gkae248)
Supplement: gkae248_Supplemental_Files [file gkae248_supplemental_files.zip › Narunsky 50 Genomes RNA Motifs Files.zip!/Publication/47-P_difficile/Figures/LRC-47-3-NC_009089_1_IGR_12_3493626_3493784.sto.pdf]

LRC-47-3-NC_009089_1_IGR_12_3493626_3493784.sto.r2r

5′-○○○○○●CAUCCUCUCAUAURUYUUUUUACAAYAUUUUAUGARRUUYGAUAURUAUYYGUUCUY●UAUUU●CAAACUUUAYU●●UAU●ACURA●●AURUU●YUAY●UA●AYUUAU●U●●U●●YAYYUAUAUUUAUCUA●U●●UUUAAA●UUAY●AU○○
